# The Potential Role of Dietary (Poly)phenols in Cardiometabolic Risk During Menopause: A Narrative Review

**DOI:** 10.3390/nu18071130

**Published:** 2026-03-31

**Authors:** Lorena Sánchez-Martínez, Rocío González-Barrio, María Jesús Periago

**Affiliations:** 1 Department of Food Technology, Food Science and Nutrition, University of Murcia, Campus Mare Nostrum, Campus de Espinardo, 30100 Murcia, Spain; lorena.sanchez14@um.es; 2 Biomedical Research Institute of Murcia Pascual Parrilla—IMIB, 30120 Murcia, Spain

**Keywords:** bioactive compounds, postmenopause, gut microbiota, insulin resistance, blood pressure, lipid profile, inflammation, endothelial cell adhesion biomarkers, oxidative stress

## Abstract

Menopause is a pivotal stage in women’s life that brings with it multiple physiological changes that significantly increase the risk of cardiometabolic diseases. (Poly)phenols are plant secondary metabolites that present several mechanisms of action that could improve human health, including the regulation of gene expression, the control of lipid metabolism, the maintenance of glucose homeostasis, a reduction in blood pressure, prebiotic effects, and antioxidant and anti-inflammatory activities. This narrative review summarizes current evidence on the main cardiometabolic risk factors associated with menopause (i.e., obesity, dyslipidemia, high blood pressure, and insulin resistance) and examines the potential of dietary strategies focused on (poly)phenol intake to mitigate these alterations. Current evidence suggests that dietary intervention based on (poly)phenol intake could be a great strategy to mitigate cardiometabolic alterations during menopause. Moreover, this review underscores the crucial need to develop personalized nutrition strategies to optimize the effectiveness of (poly)phenol-rich diets for postmenopausal women’s health, thereby alleviating the cardiometabolic risk associated with this pivotal stage of women’s lives. In addition, this work emphasizes that future research should comprehensively address the key factors involved in the main mechanisms of action of (poly)phenols in promoting health, including (poly)phenol bioavailability, the role of the gut microbiota in the colonic metabolization of these bioactive compounds, and the regulation of gene expression via nutrigenomic effects related to cardiometabolic diseases. This integrative approach will be essential for establishing evidence-based dietary recommendations for (poly)phenol intake during menopause.

## 1. Introduction

Menopause is a pivotal stage in a woman’s life that is characterized by the loss of ovarian reproductive activity due to the natural ageing process or associated with another underlying condition such as oophorectomy, a pharmacological treatment, or others [1]. As a result of this physiological process, women experience a decline in ovarian steroid hormones (i.e., estrogens and progesterone) and an increase in follicle-stimulating hormone (FSH) and bioavailable testosterone, which lead to alterations in the menstrual cycle until the definitive cessation of menstruation [2,3].

**Figure 1:**
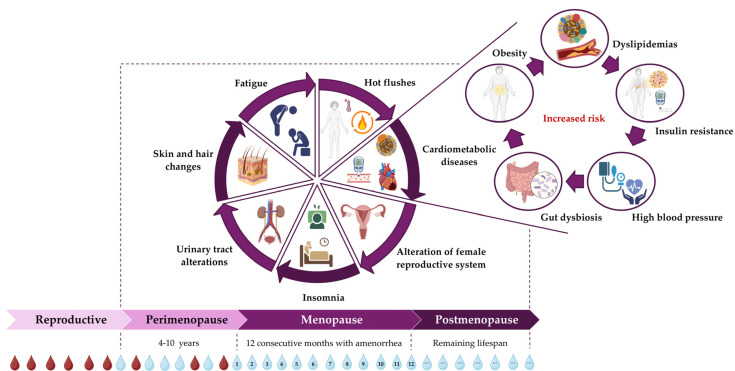
Chronological stages of the female hormonal transition, and the main clinical symptoms of perimenopause, menopause and postmenopause [4,7,19].

This hormonal transition occurs in three consecutive stages: perimenopause, menopause, and postmenopause, as shown in Figure 1. Perimenopause is the first stage, beginning with the initial change in the menstrual cycle and ending when menopause is officially established. Menopause is defined as 12 consecutive months without a period. Postmenopause is the final stage of the female reproductive cycle, occurring after menopause and continuing for the rest of a woman’s life [4,5].

It is currently unknown how the menopause stimulus triggers ovarian follicular activity. However, most research focuses on the expression of the 9q21.3 region of chromosome 9 as the main trigger for the onset of the hormonal transition toward menopause. In this region, there is a gene encoding a protein from the B2 cell lymphoma family, which is involved in cell apoptosis and whose expression is associated with premature menopause (<40 years) and early menopause stages (<45 years). Thus, its expression may be directly related to follicular depletion and therefore to the onset of menopause [6].

The age at which menopause occurs generally ranges from 48 to 52 years, influenced by factors such as lifestyle (i.e., diet, physical activity, and smoking habits), physiological factors [i.e., body mass index (BMI) or body fat and muscle mass], socioeconomic background, ethnicity, cultural beliefs, and medical history of gynecological health [3,7].

A healthy lifestyle and regular physical activity have been linked to delaying menopause and reducing symptom severity. Conversely, regular tobacco use has been connected to earlier perimenopause onset and a shorter duration of this phase [8]. Regarding anthropometric measurements, higher BMI values are associated with a later onset of perimenopause but a shorter transition period [9]. However, the impact of body composition, specifically fat content, on menopausal age remains controversial. Some researchers found that high fat mass delays the onset of perimenopause [10], while others report that high body fat levels are linked to earlier perimenopause [11]. These discrepancies may partly be explained by the fact that the FSH receptor is primarily expressed in visceral fat adipocytes [12]. Therefore, not only BMI but also body composition influences the onset of menopause.

Ethnicity also influences the duration of the menopausal transition, with studies showing that African American women experience a longer transition than Caucasian women. Likewise, socioeconomic status affects the timing of menopause, as it has been observed that women in low- or middle-income countries tend to reach menopause earlier than those in high-income countries [3,7]. This may partly be due to differences in diet quality, as it is estimated that malnourished women stop menstruating about 4 years earlier than well-nourished women [13]. Overall, the average age of menopause for healthy European women is between 50.5 and 51.2 years, although it varies considerably among individuals, from 40 to 60 years, due to the factors mentioned above [6]. 

Life expectancy for women in Europe is 84 years [14], meaning that European women spend about 40% of their lives in the postmenopausal stage. During this period, women experience physiological and metabolic changes that impact their physical, emotional, mental, and social well-being, leading to very specific symptoms that affect more than 80% of women. Additionally, it should be noted that an earlier onset of these symptoms is linked to a longer duration and greater severity [7,15]. Figure 1 displays the main clinical symptoms of menopause, which include the following:

- Hot flushes and night sweats, which appear as a sudden feeling of heat in the face, neck, and chest, along with flushing, sweating, and palpitations.
- Alterations in the female reproductive system, such as vaginal dryness and dyspareunia.
- Difficulty sleeping or dealing with insomnia.
- An increased incidence of obesity, hypertension, and type II Diabetes Mellitus (T2DM), which lead to a higher risk of cardiometabolic diseases (R-CMDs), including cardiovascular diseases (CVDs).
- Alterations of the urinary tract, such as incontinence or increased frequency and urgency.
- Mood swings that might cause depression and/or anxiety.
- Fatigue and joint pain.
- Changes in skin, hair, and nails, such as dry skin and/or thinning nails.

Among these clinical symptoms, the increase in CMDs that lead to a higher risk of CVDs is of particular interest, since it is known that until this stage of life, women have a lower incidence of these conditions compared to men. Specifically, in individuals under 54 years old, CVDs cause 22% of deaths in men and 18.5% in women. However, from the age of 55, mortality due to CVDs doubles in women, reaching 41%, while in men it is around 38.5% [16,17]. For this reason, CVDs are among the leading causes of death in postmenopausal women. This is mainly due to the cardioprotective effect of estrogen in the body, which women experience until perimenopause, a stage when estradiol levels start to decline from an average of about 100–250 pg/mL to a postmenopausal level of 10 pg/mL [3,18].

As a result of the decline in estradiol, women experience anatomical changes where they shift from a gynoid phenotype, characterized by fat accumulation in the extremities, to an android phenotype, marked by fat buildup in the abdominal area. These anatomical changes are accompanied by hormonal profile shifts, transitioning from an estrogenic profile (dominated by estradiol and progesterone) to an androgenic profile (dominated by testosterone and dehydroepiandrosterone). Additionally, physiological changes are triggered, such as alterations in lipid metabolism, an increased risk of insulin resistance, or higher blood pressure (BP).

These changes lead to a progressive increase in the risk of cardiovascular diseases (R-CVDs) during the perimenopausal period. When menopause occurs, the R-CVDs in women surpasses that of men, making CVDs the leading cause of death and illness in women. Although the severity depends on health status at menopause, factors such as age, lifestyle, environment, and culture also play a role.

Regarding the pathophysiology of CVDs, the differences between men and women in terms of anatomical structure, body composition, hormonal profile, and cardiovascular function have led to misinterpretations, with women’s R-CVDs being underestimated compared to men’s. However, over the past decade, awareness of these diseases in women has increased, and they are now considered the leading cause of morbidity and mortality in this group [20]. Additionally, the American Heart Association (AHA) has identified menopause, along with gestational diabetes and pre-eclampsia, as specific risk factors for women [21].

Plant-based foods are a major source of bioactive compounds, including terpenoids, carotenoids, and (poly)phenols, which have a wide range of effects relevant to human health [22]. Among these, (poly)phenols are secondary plant metabolites widely recognized for their antioxidant and anti-inflammatory properties, as well as their ability to regulate lipid metabolism, maintain glucose homeostasis, reduce BP, and modulate gene expression [23]. These mechanisms may help alleviate menopausal symptoms and decrease the R-CMDs (such as T2DM, dyslipidemia, and hypertension) in postmenopausal women. Therefore, dietary (poly)phenols have become a promising nutritional approach to improve overall health and quality of life in this population. However, the exact mechanisms by which these bioactive compounds reduce cardiometabolic risk are still not fully understood.

Against this background, this narrative review aims to (i) summarize the current evidence on the main cardiometabolic risk factors associated with menopause (such as obesity, dyslipidemia, high BP, and insulin resistance); (ii) evaluate the potential of dietary strategies based on (poly)phenol intake to mitigate these changes; and (iii) identify existing knowledge gaps in this field that could be addressed by future research.

## 2. Literature Survey

A thorough search of the scientific literature was conducted in PubMed, Web of Science, and Google Scholar using various combinations of the following keywords: “Menopause”, “Postmenopause”, “Menopause clinical manifestations”, “Body Composition”, “Dyslipidemias”, “Glycemic Index”, “Insulin resistance”, “Blood pressure”, “Gut microbiota”, “(Poly)phenols”, “(Poly)phenol-rich foods”, “Mediterranean Diet”, “Metabolic Diseases”, and “Cardiovascular Diseases”. The studies selected were based on their relevance to the topics covered in this review. Articles published in English between January 2004 and January 2026 were included, with a special focus on those offering insights into the menopausal transition, cardiometabolic risk, and dietary (poly)phenols, including their diversity, bioavailability, and potential cardioprotective effects.

Additionally, the reference lists of relevant articles and review papers were examined manually. The Newcastle–Ottawa Scale, commonly used to evaluate the quality of non-randomized studies in meta-analyses, was not utilized, as this manuscript is a narrative review.

## 3. Menopause Clinical Manifestations Involved in Cardiometabolic Risk

The hormonal transition to menopause involves multiple physiological, psychological, and social changes that impact women’s health and quality of life. These changes result from the interactions between neuroendocrine shifts and alterations in the reproductive endocrine axis that regulate ovarian functions, leading to a sudden decrease in estrogen levels [6]. Among the clinical signs of menopause, those related to an increase in R-CMDs are particularly significant because of their role in the morbidity and mortality of postmenopausal women, as described below.

### 3.1. Changes in Body Composition Associated with Overweight and Obesity

Postmenopausal women gain about 0.68 kg per year and have 49% more abdominal fat compared to premenopausal women [17]. In addition, these changes in body composition are characterized by a greater accumulation of visceral fat, increasing the visceral fat index from 5–8% to 15% [24]. Visceral fat is metabolically active and produces several inflammatory markers with important atherogenic properties, which can lead to a chronic state of inflammation and oxidation that significantly increases R-CMDs such as T2DM or dyslipidemias [24]. This tendency to develop obesity and accumulate fat in the abdominal area may be due to the increase in bioavailable testosterone, since this hormone favors the android phenotype over the gynoid and increases fat deposition in visceral tissues, including the heart and aorta [25,26,27]. Furthermore, the tendency toward obesity increases over the years because metabolic expenditure decreases with age, as basal metabolism and physical activity levels decline. Additionally, thermogenesis in brown adipose tissue decreases due to reduced interaction between estrogens and the sympathetic nervous system [16].

On the other hand, the states of anxiety and depression that postmenopausal women often experience could lead to excessive energy consumption associated with the intake of surplus foods and a sedentary lifestyle, which further alters body composition. Therefore, maintaining body weight within normal-weight range may help to reduce the adverse effects associated with menopause.

### 3.2. Increased Plasma Lipid Concentrations and Dyslipidemias

The hormonal transition toward menopause results in a rise in dyslipidemia cases, characterized by higher triglycerides (TGs) and low-density lipoprotein cholesterol (LDL-C) levels, along with lower high-density lipoprotein cholesterol (HDL-C) levels, as demonstrated by the Study of Women’s Health Across the Nation (SWAN) [28]. This study assessed the hormonal changes during menopause in women of various ethnicities (including Caucasian, African American, Hispanic, Japanese, and Chinese) who were not on hormone therapy. Notably, postmenopausal women experience an increase in LDL-C of about 10–20% and TGs of approximately 10–15%, which contributes to the development of atherosclerosis [6,29].

These changes in the lipid profile are primarily due to increased activity of lipoprotein lipase in adipose tissue, which catalyzes the conversion of TGs into free fatty acids for absorption and storage in adipocytes, leading to a noticeable increase in lipid reserves. In turn, excess visceral abdominal fat causes more lipolysis, resulting in a higher flow of fatty acids to the liver and increased resistance to hepatic insulin [30]. Additionally, β-oxidation of fatty acids in mitochondria decreases because of reduced interaction between estradiol and the genes involved in this process [18]. Moreover, as adipose tissue expands, there is increased production of adipokines, which contributes to chronic inflammation and decreased fat oxidation [2,6].

These changes favor the development of several cardiometabolic disorders, including metabolic syndrome (MetS), which increases by about 60% in postmenopausal women, and non-alcoholic steatohepatitis, which rises by 13.2%, among others [6,31,32,33]. Therefore, improving the lipid profile during menopause is crucial to decrease the R-CMDs associated with this stage and to enhance the health of postmenopausal women. This can be achieved through better fat quality in the diet and increased physical activity [34,35].

### 3.3. Predisposition to Insulin Resistance and Type II Diabetes

Changes in body composition characterized by overweight or obesity and the accumulation of visceral fat, along with the dyslipidemias of postmenopausal women, produce cytokines and other factors that increase peripheral insulin resistance [27]. Additionally, lower estrogen levels combined with higher bioavailable testosterone further decrease insulin sensitivity during the menopausal transition and significantly raise the risk of developing T2DM in postmenopausal women [16]. Epidemiological data actually show that the risk of impaired glucose tolerance increases by about 6% each year after menopause [29]. Moreover, women who experience menopause before age 50 are at a higher risk of developing insulin resistance than those who go through it after age 50 [36].

Insulin resistance is a well-known risk factor for MetS and CVDs, since elevated insulin levels in the bloodstream, caused by insulin resistance, contribute to increased levels of LDL-C and TGs, at the expense of HDL-C. In fact, postmenopausal women with diabetes have a mortality rate from CVDs that is 2 to 3 times higher than that of postmenopausal women without this condition [16,37]. Therefore, maintaining glucose homeostasis becomes crucial to reducing R-CMDs in postmenopausal women.

### 3.4. Increased Blood Pressure

Estrogens play a key role in controlling BP because they influence the homeostasis of the renin–angiotensin system, promote the production of nitric oxide (NO), participate in sodium metabolism, and increase the synthesis of prostaglandins in arterial endothelial cells, resulting in a vasodilator effect [6,38]. Therefore, as a result of the decreased estrogen/androgen ratio during menopause, postmenopausal women experience increased production of vasoconstrictor substances like endothelin and angiotensinogen [2,30]. Additionally, insulin resistance associated with menopause leads to higher circulating insulin levels, causing sodium and fluid retention, which can result in hypertension and potentially lead to congestive heart failure [39].

As a result, postmenopausal women have significantly higher BP than premenopausal women. In fact, epidemiological studies have shown that BP increases during menopause by approximately +2 mm Hg [40]. We must emphasize that, from a clinical perspective, this fact poses a substantial risk to women’s cardiovascular health, because each +2 mm Hg increase in systolic blood pressure (SBP) significantly raises the risk of mortality from ischemic heart disease by 7% and from stroke by 10% [41]. Therefore, managing BP during the hormonal transition to menopause could play a crucial role in reducing the risk of death from CVDs in postmenopausal women.

### 3.5. Changes in Gut Microbiota Profile That Can Lead to Dysbiosis

The gut microbiota is a complex and diverse community of trillions of microorganisms that inhabit the gastrointestinal tract and provide beneficial effects on the host’s physiology, metabolism, and immunity. This microbial ecosystem maintains a symbiotic relationship with the host, where its metabolic activity produces potentially bioactive metabolites, bioavailable hormones, and circulating cytokines necessary for human metabolism [42]. They are also involved in synthesizing vitamins and extracting energy from macromolecules that are considered non-digestible in the small intestine, such as undigestible polysaccharides, proteins, and secondary plant metabolites like (poly)phenols.

In addition, the anaerobic fermentation activity of the gut microbiota produces short-chain fatty acids (SCFAs), which are quickly absorbed and benefit the host’s health. Butyrate, acetate, and propionate are the most common SCFAs in the human large intestine, although others such as isovalerate, valerate, isocaproate, caproate, and heptanoate are also produced in smaller amounts. The main producers of these SCFAs include *Bacteroidetes*, *Firmicutes*, *Lachnospiraceae*, *Lactobacillus*, *Faecalibacterium*, and *Ruminococcus* [43].

SCFAs offer significant protection for the host’s health, both locally and systemically. In the gut, SCFAs help maintain the integrity of the intestinal epithelial barrier, increase mucus production by intestinal cells, reduce luminal pH, regulate tight junction proteins, reduce inflammation, and modulate immune cell responses. Systemically, SCFAs influence lipid metabolism and cholesterol synthesis, support the growth of bacteria with hydrolase activity that deconjugate primary bile acids, and aid in their reabsorption for cholesterol metabolism. They also play a role in glucose homeostasis by increasing gut-derived hormones like peptide-1 and peptide YY, which promote satiety. Additionally, SCFAs help regulate BP by interacting with G protein-coupled receptors, such as Gpr41 (activation causes hypotension) and Olfr78 (activation causes hypertension) [43].

The gut microbiota profile, and therefore its beneficial effects on the host’s health, depends on various factors such as age, gender, diet, environment, and/or the use of antibiotics and other drugs. The main bacterial phyla in a healthy microbial community include *Firmicutes* (64%), *Bacteroidetes* (23%), *Proteobacteria* (8%), and *Actinobacteria* (3%) [44]. Although the balance between bacterial species within these phyla—some of which can be beneficial or harmful to the host’s health—can change throughout an individual’s life in response to exogenous and endogenous factors, including menopause due to associated hormonal physiological changes.

Estrogens and gut microbiota maintain a bidirectional relationship, in which the presence of estrogens in the large intestine acts as a prebiotic, during the reproductive stage, promoting the growth and development of the estrogenic flora. Meanwhile, the estrogenic flora promotes increased estrogen levels by deconjugating the glucuronide or sulphate groups of sex steroid hormones, allowing for their enterohepatic recirculation, and thus maintaining estrogen levels in the body for longer [45,46]. Therefore, the gut microbiota is considered one of the key regulators of circulating estrogen levels [45,46]. In this context, the term ‘estrobolome’ appears, which is defined by Plottel and Blaser [47] as “*the aggregate of enteric bacterial genes whose products are capable of metabolizing oestrogens*”.

Therefore, during the hormonal transition toward menopause, due to the sudden decline in estrogen levels, women experience changes in the gut microbiota profile that lead to a decreased estrogenic flora. Specifically, it has been reported that postmenopausal women have gut dysbiosis characterized by a lower *Firmicutes*/*Bacteroidetes* ratio, a reduced abundance of the *Lachnospiraceae* family, and a significant decrease in the relative abundance of *Akkermansia muciniphila*, *Clostridium lactatifermentans*, *Escherichia coli-Shigella* spp., *Oscillibacter* spp. *strain KLE1745*, *Parabacteroides johnsonii*, and *Veillonella seminalis* [46]. Conversely, an increase in the relative abundance of the genera *Prevotella*, *Parabacteroides*, *Bacteroides*, and *Bilophila* has been observed [46,48]. Other studies have reported that the gut microbiota profile of postmenopausal women shows low levels of the main producers of SCFAs, such as *Faecalibacterium*, *Bifidobacterium*, *Alistipes*, *Ruminococcus*, and *Roseburia*, along with other beneficial bacteria like *Akkermansia muciniphila*, *Eubacterium eligens*, and *Eubacterium rectale* [49].

Furthermore, the obesity trend linked to menopause worsens gut dysbiosis by further decreasing microbial diversity and lowering the relative abundance of *Akkermansia muciniphila* [50,51]. This bacterium helps maintain the thickness of the intestinal epithelial mucosal layer, and its reduced presence can cause a leaky gut, which may allow toxins to cross the intestine and promote a proinflammatory and pro-oxidative state in the body [50]. 

Gut dysbiosis has recently been identified as a risk factor in the development of CMDs, and the *‘gut–heart’* concept has been introduced, emphasizing the importance of gut microbiota in the host’s cardiovascular health by regulating cholesterol metabolism and the conversion of choline to trimethylamine-*N*-oxide (TMAO) [42,52]. Scientific evidence has demonstrated a direct connection between the presence of specific microorganisms in the large intestine—such as *Collinsella*, *Helicobacter pylori*, *Streptococcus* spp., *Enterobacteriaceae*, and *Clostridium*—and an increase in the R-CMDs [53,54]. In fact, some of these microbes can serve as diagnostic markers for cardiovascular events like coronary artery disease [55].

Therefore, maintaining microbial diversity during menopause through the intake of prebiotics and/or probiotics is a promising strategy to reduce the negative effects associated with gut dysbiosis experienced by postmenopausal women, due to the decrease in estrogen levels and the increased tendency toward obesity during this stage of women’s lives.

## 4. Strategies to Reduce Menopause-Related Cardiometabolic Risk

Menopause management has been the focus of several studies aimed at developing strategies to reduce the adverse effects linked to this hormonal transition, which impacts women’s health and quality of life. These strategies include hormonal therapy, pharmacological treatments with drugs to alleviate symptoms, and lifestyle changes such as promoting physical activity and adopting healthy diets [6,56].

To date, hormone replacement therapies (HRTs) have been the most used strategies to reduce the adverse effects of menopause. However, recent clinical trials involving postmenopausal women reveal a link between these therapies and increased prevalence of other conditions such as cancer (i.e., breast and ovarian) or Alzheimer’s disease [57,58]. Additionally, early findings from the Women’s Health Initiative study indicated that HRT does not provide cardioprotection for all women and may raise the risk of stroke and venous thrombosis in healthy postmenopausal women over 50 years old [38,59].

Against this background, developing new strategies to mitigate menopause-related adverse effects in general and specifically to reduce the R-CMDs using natural compounds such as plant foods or plant extracts—without harming women’s health—becomes a major challenge for the scientific community. In this context, the scientific literature is increasingly reviewing studies aimed at exploring how the R-CMDs in postmenopausal women can be managed through various dietary approaches. These include the development of low-fat and/or low-carbohydrate diets to improve glucose and lipid metabolism [60,61,62], low-calorie diets focused on weight loss to enhance body composition and anthropometric measurements [63], and plant-based diets for their antioxidant and anti-inflammatory properties to fight oxidative stress and chronic inflammation [64].

The effects of established dietary patterns such as the Mediterranean, Central European, and/or Paleolithic diets have also been evaluated. For example, Bajerska et al. [65] compared the effects of a Mediterranean-style diet with those of a Central European-style diet on the cardiometabolic health of postmenopausal women. After 16 weeks of dietary intervention, they observed improvements in women’s health, including reductions in body weight, the Homeostatic Model Assessment of insulin resistance index (HOMA-IR), total cholesterol (T-C), and TGs, for both diets. The Paleolithic-style diet has also been tested in postmenopausal women and linked to fat loss and decreased TGs [66]. Similarly, other clinical trials have been conducted to evaluate the effectiveness of dietary recommendations from public institutions such as the AHA. In this context, Senechal et al. [67] reported significant improvements in the lipid profile of postmenopausal women, characterized by notable reductions in T-C, LDL-C, and TGs after a 12-week dietary intervention.

It should be noted that these research findings suggest plant foods as potential allies in combating the R-CMDs in postmenopausal women, due to their high content of bioactive compounds such as carotenoids, terpenes, and (poly)phenols [68,69]. Carotenoids are known for their antioxidant capacity and hypocholesterolemic effects [70], and a previous dietary intervention reported a significant improvement in lipid profiles (with a decrease in LDL-C levels) as well as in inflammatory status [due to reduced C-reactive protein (CRP) levels and increased adiponectin levels] after supplementing the diet of peri- and postmenopausal women with carotenoid-rich foods (415 mg of total carotenoids per week) for 4 weeks [71]. Other bioactive compounds, such as terpenes, possess strong anti-inflammatory activity, and their intake has been associated with improvements in inflammatory diseases, including CMDs [72,73]. Regarding (poly)phenols, they are the most abundant secondary plant metabolites in plant foods and are recognized for their antioxidant, vasodilator, and gene expression-regulating properties [74], making them promising molecules for reducing the R-CBMs in postmenopausal women. For example, Chai et al. [75] reported an improvement in the cardiovascular health of postmenopausal women, evidenced by a significant decrease in T-C and LDL-C levels after daily intake of dried apples for 3 months.

Although increasing plant foods in the diet leads to a higher intake of various bioactive compounds, as mentioned above, this review focuses on the role of dietary (poly)phenols as a natural strategy to reduce the R-CMDs in postmenopausal women.

## 5. (Poly)phenols: Structural Diversity, Absorption and Metabolism

(Poly)phenols are secondary plant metabolites found widely in plant-based foods because they are crucial for plant growth and development. They are mainly produced by plants as a defence against biotic agents such as pathogens (like viruses and bacteria) and herbivores, as well as abiotic stresses like ultraviolet radiation and water scarcity [76,77]. (Poly)phenols are primarily synthesized in plants through two metabolic pathways: the shikimic acid pathway (which mainly produces phenylpropanoids) and the acetic acid pathway (which creates simple phenols) [44]. They can also be produced, to a lesser degree, by fungi and bacteria via the malic acid pathway. Phenylalanine is the main precursor of (poly)phenols, but as an essential amino acid for animals, they cannot produce (poly)phenols themselves and must obtain them through their diet [78]. 

**Figure 2:**
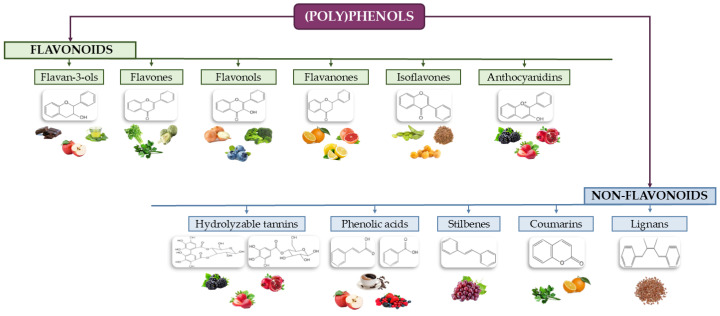
Structural diversity of (poly)phenols and their distribution in foods [77].

More than 8000 compounds have been identified as part of this extensive family of bioactive compounds. All of these contain at least one phenol group at the structural level, which is formed by a hydroxylated benzene ring. As shown in Figure 2, (poly)phenols can be classified based on their structure into two main groups: flavonoids and non-flavonoids. These groups are further divided into subclasses based on the number of phenolic rings they contain and the structural elements connecting them. Their content in various plant foods varies depending on factors such as botanical family, cultivar, agricultural conditions, and processing methods. Flavonoids include flavan-3-ols, present in cocoa beans, green tea, apples, and cereals; flavonols, found in onions, berries, broccoli, and green tea; flavanones, the primary (poly)phenols in citrus fruits (orange, grapefruit, lemon, and lime); isoflavones, present in soybeans and their products; flavones, found in citrus fruits, cereal grains, herbs, and artichoke; and anthocyanins, present in various berries (raspberries, blackcurrant, blueberries, strawberries and cherries), red grapes, radish, pomegranate, and red plum. Non-flavonoids include phenolic acids such as cinnamic acids (found in coffee, blueberry, apple, and apricot) and benzoic acids (present in raspberry, tea, cloudberry, and chestnut); hydrolysable tannins like ellagitannins (found in berries, pomegranate, walnut, and green tea) and gallotannins (found in mango, chestnut, and red sword bean); stilbenes (found in grape and red wine); coumarins (present in citrus, parsley, and celery); and lignans (found in flaxseed and sesame seeds) [77].

In recent years, the intake of these phytochemicals has been associated with beneficial effects on human health and a reduction in cancer and other non-communicable diseases, such as CMDs, mainly due to their antioxidant, anti-inflammatory, vasodilator, immune response-stimulating, and gene expression-regulating properties [74,79]. However, the extent of the benefits from consuming these bioactive compounds depends on several factors, such as the amount of (poly)phenols in the diet, the structure of the ingested compounds, their bioaccessibility and bioavailability in the small intestine, and how they are metabolized by gut microbiota activity [77]. 

Generally, the total intake of (poly)phenols is about 1 g per day for people who eat several servings of fruits and vegetables each day [80,81]. Specifically, in Spain, the average intake has been estimated at 820 mg daily, with about 440 mg from flavonoids and around 300 mg from phenolic acids [82]. However, a study by the research team based on the ENIDE survey shows that the average intake of phenolic compounds in the Spanish diet reaches 1.36 g per person daily, with plant-based beverages, legumes, and fruits as the main sources of these compounds [83].

Because of the structural differences of (poly)phenols, their bioavailability varies greatly, with estimated values of 0.30% for anthocyanins and 43% for isoflavones. It is established that the bioavailability of flavonoids follows the order isoflavones > flavan-3-ols > flavanones > flavonols > anthocyanins [84]. The absorption and metabolism of (poly)phenols are affected by several factors, such as their hydrophilic and lipophilic properties, binding to sugars, or the level of polymerisation, which cause some compounds to be absorbed faster than others, through different mechanisms (passive diffusion or transport proteins), or require prior metabolism by gut microbiota [77,85]. Moreover, factors such as the food matrix, binding to proteins or polysaccharides of the cell wall, and fat content can hinder the bioaccessibility and absorption of (poly)phenols in the small intestine. On the other hand, bioavailability tends to be faster in liquid foods [77,86].

**Figure 3:**
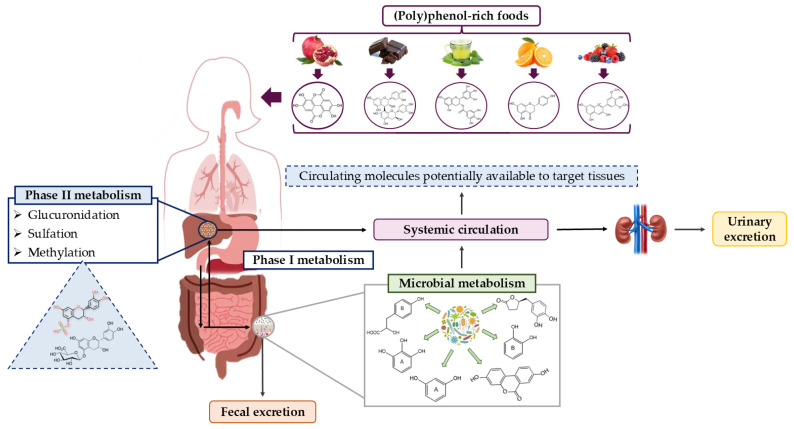
Pathway of (poly)phenol metabolism in humans [87,89].

During digestion, a small portion of (poly)phenols from the diet is absorbed in the small intestine after glycoside hydrolysis by brush border enzymes such as lactase phlorizin hydrolase and cytosolic β-glucosidase, which release the aglycones. In this form, they can enter the enterocyte via passive diffusion, although transport by membrane proteins has also been described. These compounds reach the liver, and before entering the bloodstream, their metabolism follows a common pathway involving conjugation and reconjugation reactions mediated by enzymes like sulphotransferase, UDP-glucuronosyltransferase, and catechol-*O*-methyltransferase (Figure 3). Notably, some of these phenolic metabolites can return to the digestive lumen through enterohepatic circulation [87]. However, most (poly)phenols are not absorbed in the small intestine and instead reach the large intestine intact, where they can exert a prebiotic effect by being metabolized by gut microbiota. This process modulates their profile toward a healthier state and produces small phenolic metabolites that can be absorbed [88,89]. These phenolic metabolites are more active than their precursors because they are absorbed more easily and have a longer half-life, thereby enhancing the beneficial effects of these bioactive compounds in the body [77,90]. It is important to note that the type of microbial transformation depends on the structure of the (poly)phenol (whether flavonoids or non-flavonoids), their degree of polymerization, the spatial configuration of the compound, and the composition of the host’s gut microbiota [86,89].

Generally, (poly)phenols and their phenolic metabolites tend to have a higher affinity for the aqueous environments in the body because of their hydrophilic nature, but they do not circulate freely in the bloodstream. Therefore, once absorbed, plasma albumin is considered the main carrier responsible for binding and transporting phenolic compounds in the blood [80]. However, lipoproteins also play a role in transporting (poly)phenols, as studies in humans have shown that (poly)phenols can bind to lipid structures such as LDL lipoproteins and chylomicrons, thereby exerting physiological effects [91,92]. The highest concentrations of these compounds in blood usually occur within the first 2 h after ingestion, reflecting the initial phase of absorption in the small intestine. Nevertheless, a second, often significant phase of circulating phenolic metabolites occurs later (around 6–10 h after ingestion) due to microbial metabolism of non-absorbed (poly)phenols. These microbial-derived phenolic metabolites can stay in circulation for extended periods, maintaining detectable levels for up to 24 h and possibly supporting more prolonged physiological effects. Consistent with this extended systemic presence, their detection in urine can persist for up to 24 h (e.g., flavonols), suggesting that urinary excretion may be a better indicator of overall (poly)phenol bioavailability [77,93,94]. Finally, (poly)phenols, along with hepatic and microbial metabolites not absorbed during digestion along the gut, are expelled through feces.

## 6. (Poly)phenols and Their Beneficial Effects on Cardiometabolic Diseases

Extensive scientific evidence shows a positive link between consuming (poly)phenol-rich foods (PP-rich foods), such as fruits, vegetables, whole grains, and their derived products like juices, and a lower risk of non-communicable diseases like CMDs (i.e., hypertension, diabetes, dyslipidemias, and/or CVDs).

Several epidemiological studies have shown that the intake of (poly)phenols significantly reduces morbidity and mortality from all causes, including CVDs. For example, in the PREDIMED study, an inverse correlation was observed between PP-rich diets, such as the Mediterranean diet (MD), and mortality in subjects with high R-CVDs. Specifically, the researchers noted a 37% reduction in all-cause mortality when comparing the highest (1235 mg/day) with the lowest (483 mg/day) quintiles of (poly)phenol intake [82]. Similarly, the “*Seguimiento Universidad de Navarra*” (SUN) cohort study [95], conducted in healthy middle-aged Spanish adults, found that after 10 years of follow-up, participants with high flavonoid intake (highest quintile) had a 47% lower incidence of cardiovascular events compared to those with low flavonoid intake (lowest quintile). Additionally, they suggested that foods such as fruits like apples and oranges, dark chocolate, nuts, and green tea—being the most consumed by the Spanish population and major contributors to dietary (poly)phenol intake—could be responsible for this cardioprotective effect, as these foods provide a wide variety of flavonoids and non-flavonoid compounds [95].

Although *in vitro* assays suggest that the beneficial effects of (poly)phenols in reducing the R-CMDs are due to their antioxidant, anti-inflammatory, vasodilator, and immune response-regulating properties [96], in vivo studies have shown that they can also affect the gut microbiota by promoting beneficial bacteria and inhibiting harmful ones. Additionally, they can regulate gene expression and reverse pathological processes related to CMDs [97,98]. 

**Figure 4:**
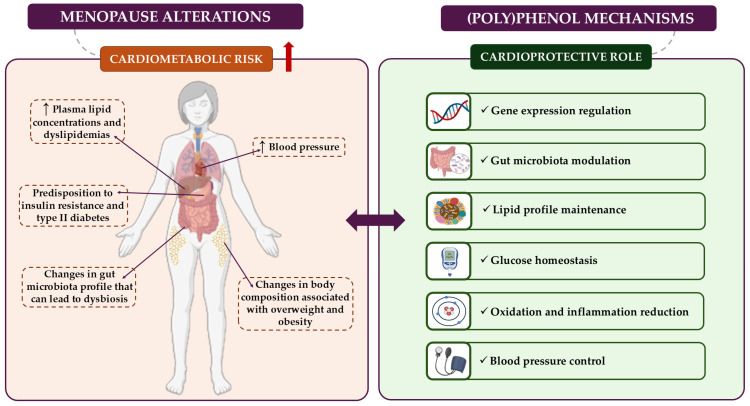
The main menopause-related alterations involved in cardiometabolic risk and the possible mechanisms by which (poly)phenols could alleviate it.

Against this background, several clinical trials involving foods or extracts rich in (poly)phenols are documented in scientific literature, aiming to evaluate their cardioprotective effects on human health. The results from these studies show that the main benefits of consuming PP-rich products for cardiovascular health include improvements in body composition and anthropometric measures (e.g., BMI, fat index, and waist–hip ratio) [99]; better control of BP; enhanced glucose metabolism (such as fasting glucose and insulin levels, as well as the HOMA-IR index) [100]; improved lipid profiles (including HDL-C, LDL-C, and TGs) [101]; a reduction in cell adhesion biomarkers [e.g., soluble vascular cell adhesion molecule 1 (sVCAM-1) and soluble intercellular adhesion molecule 1 (sICAM-1)]; the modulation of inflammatory biomarkers [including tumour necrosis factor-α (TNF-α) interleukin 6 (IL-6) and IL-1]; a decrease in oxidative stress biomarkers [such as thiobarbituric acid reactive substances (TBARs) and oxidized LDL (LDL_ox_)]; the influence on the gut microbiota profile [102]; and the regulation of gene expression [103]. These findings suggest that PP-rich foods operate through multiple mechanisms of action that could reduce cardiometabolic risk during menopause (Figure 4).

### 6.1. Effect of (Poly)phenols on Body Composition and Anthropometric Measurements

Several clinical trials have shown that (poly)phenol intake improves body composition and may influence body weight regulation mechanisms. These include reducing lipid and carbohydrate absorption by inhibiting digestive enzymes like pancreatic lipase [99], increasing energy expenditure through enhanced basal metabolism [99], and suppressing appetite by inhibiting the ghrelin hormone while promoting leptin and adiponectin hormones [104]. A recent meta-analysis assessing the effectiveness of dietary (poly)phenols in managing obesity found that (poly)phenol intake could decrease body weight (−0.36 kg), BMI (−0.13 kg/m^2^), and W/H ratio (−0.30 cm) [99]. These results indicate that (poly)phenol consumption may help control body weight during menopause.

Furthermore, after comparing different methods of (poly)phenol administration, the researchers concluded that consuming (poly)phenols from whole foods such as fruits and vegetables is more effective and lasting over time than taking (poly)phenol extracts. We must emphasize that this is very important for clinical purposes, as effective weight loss needs to be slow and sustainable to have a meaningful impact on the body [99]. 

In this line, Katanasaka et al. [105] reported that consuming catechin-rich green tea significantly improved body composition in obese individuals by reducing body weight, BMI, and waist circumference. Abedini et al. [106] observed a notable decrease in BMI among women with polycystic ovary syndrome after drinking 45 mL/day of pomegranate juice for 8 weeks. However, García-Yu et al. [107] noted a favourable, though not statistically significant, decrease in BMI in postmenopausal women after consuming 10 g of 99% cocoa dark chocolate.

### 6.2. Impact of (Poly)phenols on Lipid Metabolism

The improvement in the lipid profile after consuming (poly)phenols, indicated by a decrease in plasma LDL-C and/or TG levels along with an increase in HDL-C, has been documented in various clinical trials involving both healthy individuals and those with metabolic disorders such as dyslipidemias [104,108,109]. Specifically, it is estimated that taking (poly)phenols can lower LDL-C by −5.4 mg/dL, TGs by about −10.12 mg/dL, and T-C by −5.11 mg/dL [110]. From a clinical perspective, this adjustment of the lipid profile is very important because every 1% reduction in cholesterol levels is linked to a 2% decrease in cardiovascular events [110].

Scientific research shows that the main ways (poly)phenols regulate lipid metabolism and help prevent dyslipidemia include inhibiting pancreatic lipase, which decreases fat absorption and reduces the production of TG-rich lipoproteins in liver cells [101]; protecting LDL-C from oxidative stress by scavenging free radicals and binding to LDL-C [111]; increasing insulin sensitivity by preventing the conversion of excess glucose into TGs [112]; improving gut microbiota composition [113]; and regulating gene expression related to adipocyte differentiation, adipogenesis, and lipolysis [114].

In this context, Toaldo et al. [115] have shown that consuming grape juice significantly reduces TBARs, which are an indicator of the body’s lipid peroxidation levels and are directly associated with dyslipidemias and R-CMDs. Similarly, eating cocoa and cocoa-rich products has been linked to improvements in the lipid profile, including higher HDL-C and lower LDL-C, thanks to the antioxidant activity of flavan-3-ols, which can decrease cellular lipotoxicity and enhance lipid metabolism [116]. These findings suggest that consuming (poly)phenols could improve the lipid profile, thereby reducing the risk of arteriosclerosis.

### 6.3. Influence of (Poly)phenols on Glucose Metabolism and Insulin Resistance

Research on the mechanisms of action involved in the anti-diabetic properties of (poly)phenols suggests that improvements in glucose homeostasis result from the reduction in postprandial glucose, limitations in glucose transport, the involvement of insulin signalling, and the protection of insulin-secreting pancreatic β cells against oxidative damage [100,117,118]. 

In particular, (poly)phenols inhibit SGLT1, the main glucose transporter in the brush border of the small intestine that moves D-glucose from the intestinal lumen into the enterocyte. (Poly)phenols can also affect SGLT2, which is involved in kidney glucose reabsorption [100]. Additionally, the antioxidant and anti-inflammatory effects of (poly)phenols prevent oxidative damage to pancreatic β cells, helping to maintain insulin secretion [112,117]. Furthermore, (poly)phenols can stimulate NO production by activating the endothelial PI3K/Akt/NO synthase pathway, promoting GLUT4 translocation and increasing glucose uptake [119], which significantly improves insulin resistance and lowers the risk of T2DM. Specifically, it has been estimated that consuming (poly)phenols can reduce fasting blood glucose by about −1.67 mg/dL [110].

However, the effectiveness of (poly)phenols in inhibiting glucose transporter activity and protecting pancreatic β cells from oxidative damage largely depends on their chemical structure, which causes it to vary across different classes. In this context, flavan-3-ols and anthocyanins are among the most promising candidates for improving insulin resistance, while flavonols have shown only a modest hypoglycemic effect [112].

In this context, it has been shown that consuming flavan-3-ol-rich foods like cocoa and green tea can help maintain and improve glucose balance. It is important to note that some cocoa flavan-3-ols may act directly on insulin-sensitive tissues through their insulin-like activity or indirectly by regulating key proteins in the insulin signalling pathway, thus helping prevent T2DM [116,120]. Additionally, proanthocyanidins in green tea have been reported to improve body composition and glucose metabolism by inhibiting pancreatic lipase activity, which decreases the absorption of lipids and carbohydrates in the intestine [105]. Furthermore, consuming foods rich in anthocyanins, such as pomegranate juice, has also been shown to benefit T2DM management by lowering postprandial glucose levels and increasing the production of antioxidant enzymes like paraoxonase-1 [121]. Therefore, consuming PP-rich foods could be a promising strategy for reducing glucose intolerance and improving insulin resistance.

### 6.4. Impact of (Poly)phenols on Blood Pressure

(Poly)phenols can lower BP by increasing the bioavailability of NO through promoting the expression of endothelial NO synthase. They also inhibit the expression of angiotensin-converting enzyme and phosphodiesterase. NO has a hypotensive effect by enhancing the production of vascular relaxation factors such as prostacyclin and reducing the synthesis of vasoconstrictor compounds like endothelin-1 in vascular endothelial cells [122,123,124].

A recent meta-analysis assessed the effectiveness of (poly)phenols on cardiometabolic health and found that these bioactive compounds can lower SBP by −3.53 mm Hg and diastolic blood pressure (DBP) by −1.41 mm Hg [110]. This offers a significant cardioprotective benefit because an overall reduction of −5 mm Hg in SBP and −2 mm Hg in DBP leads to a 13% and 11.5% decrease in stroke risk, respectively, resulting in a notable reduction in CVD mortality [125]. Additionally, the meta-analysis results showed that PP-rich foods caused a significantly greater reduction in BP than (poly)phenol extracts.

In this regard, several clinical trials have reported notable changes in SBP or DBP following dietary (poly)phenol supplementation. For example, Johnson et al. [126] observed a significant 5.1% reduction in SBP and a 6.3% reduction in DBP after consuming 22 g of lyophilized cranberry powder for 8 weeks, highlighting the vasodilator potential of cranberry (poly)phenols. Conversely, Valls et al. [127] reported a significant improvement in BP after drinking 500 mL of orange juice, which they linked to the ability of flavanones to decrease the expression of key genes associated with hypertension. Similarly, Okamoto et al. [128] documented a significant reduction in SBP (−8 mm Hg) and DBP (−4 mm Hg) following the intake of 17 g of cocoa over 3 days. These results suggest that both SBP and DBP can be influenced by the consumption of different PP-rich sources.

### 6.5. Effect of (Poly)phenols on Oxidative Stress, Inflammation and Cell Adhesion Biomarkers

(Poly)phenols can prevent oxidative damage by scavenging free radicals—either by donating a hydrogen atom or an electron—and by inhibiting lipid peroxidation. They also promote the expression of antioxidant genes through the activation of endogenous antioxidant signalling pathways [129]. However, the antioxidant capacity of (poly)phenols and their beneficial effects largely depend on their structure, especially the number and position of hydroxyl groups and any modifications during human metabolism. A greater number of hydroxyl groups results in a stronger antioxidant effect, and the ortho- and/or para-positions of these groups on the benzoic ring have a more significant impact than other positions [130]. While other structural factors are involved, such as the distance between the carbonyl group and the aromatic ring, greater distances tend to enhance the antioxidant effect of (poly)phenols.

However, it should be considered that when (poly)phenols act as reducing agents or lose an electron, the molecule itself becomes a radical. Its interaction with transition metals can lead to the formation of pro-oxidants, which at high levels may harm human health [130]. Therefore, taking supplements with high concentrations of (poly)phenols could be risky, while getting (poly)phenols from food remains a safer approach.

Potential anti-inflammatory mechanisms have also been described for (poly)phenols, such as promoting the synthesis of anti-inflammatory cytokines (e.g., adiponectin) or decreasing levels of proinflammatory molecules (e.g., TNF-α) and endothelial cell adhesion molecules (e.g., sICAM-1 and sVCAM-1), by regulating cell signalling pathways including nuclear factor kappa B (NF-κB) and activator protein 1 (AP-1) [130,131].

For this reason, several clinical trials have examined the effect of dietary supplementation with PP-rich foods on cell adhesion, inflammatory, and oxidative stress biomarkers. In this context, Basu et al. [132] evaluated the activity of anthocyanins in enhancing systemic antioxidant capacity and reducing inflammatory status after strawberry consumption, reporting a decrease in sVCAM-1 and TNF-α linked to an increase in serum antioxidant capacity following PP-rich food intake. Other studies have shown that long-term (poly)phenol intake can also improve oxidative stress by lowering TBARs levels. For example, Kardum et al. [133] observed a positive effect on cellular oxidative damage associated with improved activity of cellular antioxidant enzymes (i.e., SOD and GPx), resulting in a significant decrease in TBARs after consuming chokeberry juice for 3 months in healthy women. Similarly, Sánchez-Martínez et al. [134] demonstrated that consuming PP-rich foods, such as dark chocolate, green tea, and fruit juice, led to a significant reduction in TBAR levels in postmenopausal women after 2 months. Hence, the chronic consumption of PP-rich foods could alleviate the R-CMDs by reducing inflammation and oxidative stress.

### 6.6. Impact of (Poly)phenols on Gut Microbiota Profile

The relationship between (poly)phenols and gut microbiota is bidirectional. The presence of (poly)phenols in the large intestine acts as a prebiotic and influences the host’s gut microbiota profile by regulating the balance of beneficial and harmful bacteria [135]. Meanwhile, gut microbiota also plays a crucial role in the biotransformation of (poly)phenols during human metabolism, shaping the type and amount of phenolic metabolites produced. This process affects their bioavailability and the potential beneficial effects that (poly)phenols may have on human health [136,137]. 

There is sufficient evidence supporting the positive relationship between the intake of (poly)phenols and improvements in human health by modulating gut microbiota into a healthier composition, which can release metabolites and hormones into the bloodstream [44,138,139]. Among the main food sources of (poly)phenols with prebiotic effects, dark chocolate, green tea, pomegranate, oranges, and berries are prominent [140].

In this context, cocoa (poly)phenol intake has been associated with an increase in *Bifidobacteria* and *Lactobacilli*, whose presence in the large intestine is linked to a stronger immune system and a reduction in CRP levels [138]. Additionally, evidence indicates that cocoa consumption is connected to a rise in SCFA-producing microorganisms [141,142]. Similarly, eating pomegranates and berries has been linked to higher levels of *Bifidobacteria*, *Lactobacilli*, and *Akkermansia*, which provide cardioprotective effects by enhancing the intestinal barrier and decreasing toxin release into the bloodstream [143,144,145]. Meanwhile, green tea (poly)phenols have been shown to inhibit the growth of harmful bacteria in the large intestine, such as *Clostridium perfringens* and *Clostridium difficile* [137,146,147]. Therefore, reducing harmful bacteria in the large intestine can promote overall health and significantly lower the risk of developing CMDs [148,149,150].

### 6.7. Role of (Poly)phenols in Regulating Gene Expression

The ability of (poly)phenols to regulate gene expression and reverse pathological processes linked to non-communicable diseases has recently been recognized as a key mechanism behind the beneficial effects of (poly)phenol intake in reducing the R-CMDs [129,151,152]. (Poly)phenols can increase the expression of genes that encode metabolic proteins with cardioprotective effects while simultaneously decreasing the expression of genes involved in oxidative stress, inflammation, cell adhesion, mobility, immune response, and cell signalling—factors that contribute to adverse cardiometabolic events. These genetic modifications may result from the interaction of (poly)phenols with signalling cascades and/or with epigenetic factors such as miRNAs, and they have been described for most classes of (poly)phenols [153,154,155]. 

Some authors have reported significant regulation of inflammatory biomarker genes, including CCL26, CX3CR1, CXCL17, C-C motif chemokine 3, IL-1β, and TNF-α, following the consumption of 500 mL of orange juice or a control drink containing 242 mg of hesperidin in healthy men. Similar effects were observed after supplementation with a resveratrol-rich grape extract in hypertensive diabetic men [156,157]. Specifically, Barrera-Reyes et al. [158] noted changes in the expression of 98 genes involved in reducing ROS, modulating calcium levels, and regulating the inflammatory response following intake of PP-rich cocoa powder [600 mg (poly)phenols, including 94 (−)-epicatechin] in healthy young adults. Therefore, different (poly)phenols may influence various metabolic pathways involved in R-CMDs.

## 7. Limitations

This review has several limitations to consider when interpreting the evidence. First, it was conducted as a narrative review; thus, the study selection process did not adhere to a fully systematic method. Although multiple scientific databases were consulted, the lack of predefined inclusion and exclusion criteria and a structured selection strategy may introduce bias and reduce the review’s reproducibility. Second, the current evidence on the cardiometabolic benefits of dietary (poly)phenols in postmenopausal women is highly varied. The available studies include epidemiological research and clinical trials with different designs, varying in populations, interventions, outcomes, and methods. This variation makes it difficult to compare results directly and draw consistent conclusions.

Another key limitation concerns the bioavailability and metabolism of (poly)phenols. These compounds can vary widely depending on their chemical structure, the food matrix in which they are consumed, individual metabolic differences, and the composition and activity of the gut microbiota. Such factors can significantly affect the extent of physiological effects observed and are not always properly controlled or reported in human studies. Additionally, many clinical trials involved relatively small sample sizes and short intervention durations. These limitations may decrease statistical power, limit the generalizability of results, and hinder the evaluation of long-term cardiometabolic outcomes.

Finally, lifestyle-related factors—such as overall diet quality, physical activity, and socioeconomic factors—may serve as potential confounders and are not consistently considered in the available literature.

Taken together, these limitations emphasize the need for well-designed, sufficiently powered randomized controlled trials and mechanistic studies to better understand the role of PP-rich diets in lowering cardiometabolic risk during the postmenopausal period.

## 8. Future Research and Perspectives

This narrative review highlights menopause as a crucial stage in a woman’s life, characterized by significant hormonal and physiological changes that substantially increase cardiometabolic risk. Evidence indicates that these changes are associated with increased body fat—particularly around the abdomen—dyslipidemias, insulin resistance, elevated BP, and gut dysbiosis, all of which contribute to a higher prevalence of cardiometabolic diseases in postmenopausal women.

This review highlights the potential of (poly)phenol intake as a promising dietary approach to reduce cardiometabolic risk during menopause. Growing evidence suggests that (poly)phenols may provide benefits through multiple mechanisms, such as controlling BP, regulating glucose and lipid metabolism, fighting obesity, and supporting a healthy gut microbiota through prebiotic effects. Additionally, new data show that (poly)phenols can influence gene expression via nutrigenomic pathways, potentially reversing or lessening processes linked to cardiometabolic diseases. However, the existing evidence is limited by its variability. Much of it comes from *in vitro* and animal studies, and clinical results are inconsistent, mainly because of differences in study design, including sample size, intervention length, (poly)phenol dosage, and menopausal status. Therefore, well-designed, long-term randomized controlled trials with sufficient sample sizes and careful control of confounding factors are necessary to confirm the cardiometabolic benefits of (poly)phenols in postmenopausal women.

Furthermore, additional research is needed to clarify the complex interactions between dietary (poly)phenols and their metabolites, gut microbiota composition, and host gene expression, as these interactions may be key to their cardiometabolic effects. Specifically, integrative approaches using multi-omics technologies—such as metabolomics, metagenomics, and nutrigenomics—will be crucial for advancing mechanistic understanding. Additionally, adopting personalized nutrition strategies that consider menopausal stage, (poly)phenol metabotypes, gut microbiota profile, and genetic background could improve the success of dietary interventions. Moreover, future studies should focus on evaluating plant-based dietary patterns rich in (poly)phenols rather than isolated compounds to better reflect real-world eating habits. These investigations will be vital for optimizing the effectiveness of PP-rich diets and establishing strong, evidence-based dietary guidelines aimed at lowering cardiometabolic risk in postmenopausal women.

## Data Availability

No new data were created or analyzed in this study.

## Abbreviations

The following abbreviations are used in this manuscript:

AHA	American Heart Association
AP-1	Activator Protein 1
BMI	Body Mass Index
BP	Blood Pressure
CHD	Coronary Heart Disease
CRP	C-reactive protein
CVDs	Cardiovascular Diseases
DBP	Diastolic Blood Pressure
FSH	Follicle Stimulating Hormone
HDL-C	High-Density Lipoprotein Cholesterol
HOMA-IR index	Homeostatic Model Assessment of insulin resistance index
HRT	Hormone Replacement Therapy
IL	Interleukin
LDL-C	Low-Density Lipoprotein Cholesterol
LDLox	Oxidized LDL
MD	Mediterranean diet
MetS	Metabolic Syndrome
NF-KB	Nuclear Factor kappa beta
NO	Nitric Oxide
R-CMDs	Risk of Cardiometabolic Diseases
SBP	Systolic Blood Pressure
SCFAs	Short-Chain Fatty Acids
sICAM-1	soluble Intercellular Adhesion Molecule 1
SUN	Seguimiento Universidad de Navarra
sVCAM-1	soluble Vascular Cell Adhesion Molecule 1
SWAN	Study of Women’s Health Across the Nation
T2DM	Type II Diabetes Mellitus
TBARs	Thiobarbituric Acid Reacting Substances
T-C	Total cholesterol
TGs	Triglycerides
TMAO	Trimethylamine-N-oxide
TNF-α	Tumour Necrosis Factor-α
